# Cell-autonomous programming of rat adipose tissue insulin signalling proteins by maternal nutrition

**DOI:** 10.1007/s00125-016-3905-8

**Published:** 2016-03-10

**Authors:** Malgorzata S. Martin-Gronert, Denise S. Fernandez-Twinn, Martin Bushell, Kenneth Siddle, Susan E. Ozanne

**Affiliations:** 1 University of Cambridge Metabolic Research Laboratories and MRC Metabolic Diseases Unit, Wellcome Trust—MRC Institute of Metabolic Science, Addenbrooke’s Hospital, Box 289, Cambridge, CB2 OQQ UK; 2 MRC Toxicology Unit, University of Leicester, Hodgkin Building, Leicester, UK

**Keywords:** Adipose tissue, Cell-autonomous mechanisms, Developmental programming, Insulin signalling, Low birthweight, Maternal diet, Protein restriction, Type 2 diabetes

## Abstract

**Aims/hypothesis:**

Individuals with a low birthweight have an increased risk of developing type 2 diabetes mellitus in adulthood. This is associated with peripheral insulin resistance. Here, we aimed to determine whether changes in insulin signalling proteins in white adipose tissue (WAT) can be detected prior to the onset of impaired glucose tolerance, determine whether these changes are cell-autonomous and identify the underlying mechanisms involved.

**Methods:**

Fourteen-month-old male rat offspring born to dams fed a standard protein (20%) diet or a low (8%) protein diet throughout gestation and lactation were studied. Fat distribution and adipocyte size were determined. Protein content and mRNA expression of key insulin signalling molecules were analysed in epididymal WAT and in pre-adipocytes that had undergone in vitro differentiation.

**Results:**

The offspring of low protein fed dams (LP offspring) had reduced visceral WAT mass, altered fat distribution and a higher percentage of small adipocytes in epididymal WAT. This was associated with reduced levels of IRS1, PI3K p110β, Akt1 and PKCζ proteins and of phospho-Akt Ser473. Corresponding mRNA transcript levels were unchanged. Similarly, in vitro differentiated adipocytes from LP offspring showed reduced protein levels of IRβ, IRS1, PI3K p85α and p110β subunits, and Akt1. Levels of Akt Ser473 and IRS1 Tyr612 phosphorylation were reduced, while IRS1 Ser307 phosphorylation was increased.

**Conclusions/interpretation:**

Maternal protein restriction during gestation and lactation changes the distribution and morphology of WAT and reduces the levels of key insulin signalling proteins in the male offspring. This phenotype is retained in in vitro differentiated adipocytes, suggesting that programming occurs via cell-autonomous mechanism(s).

## Introduction

Suboptimal nutrition during fetal and early postnatal life can have profound and lifelong detrimental effects on the health of an individual. Undernutrition in fetal life, which leads to intrauterine growth restriction (IUGR) and a low birthweight (LBW), is associated with an increased risk of developing multiorgan metabolic pathologies such as type 2 diabetes mellitus and the metabolic syndrome. The molecular mechanisms through which early nutrition permanently alters the phenotype of an organism and programmes its long-term metabolic health are slowly becoming defined.

Animal models have proven invaluable for studying the mechanisms linking early nutrition and type 2 diabetes risk [1]. In this context, we and others have used a rat model of maternal protein restriction. The offspring of low protein fed dams (LP offspring) share phenotypic similarities with LBW humans, including the development of insulin resistance in both skeletal muscle and white adipose tissue (WAT) and age-dependent loss of glucose tolerance that lead to the development of type 2 diabetes [2, 3]. Prior to the development of whole body insulin resistance, downregulation of key insulin signalling proteins such as insulin receptor substrate-1 (IRS1), glucose transporter type 4 (GLUT4), and the p110β catalytic subunit and p85α regulatory subunit of phosphatidylinositol 3-kinase (PI3K) have been observed in skeletal muscle and WAT of both LBW men and LP offspring rats [2, 4–6]. These changes were detected in 15-month-old LP offspring rats, which had already developed impaired glucose tolerance.

Changes in the levels of key insulin signalling proteins were not accompanied by alterations in the levels of corresponding mRNAs, indicating that post-transcriptional regulatory mechanism(s) are involved. Recently, microRNAs (miRNAs) have emerged as important programming factors that can regulate gene expression by modulating mRNA translation without altering mRNA levels [7, 8]. Other post-transcriptional regulatory mechanisms such as protein degradation have also been implicated in pathogenesis of type 2 diabetes [9].

In recent years, our understanding of the importance of WAT in regulating whole body metabolic homeostasis has increased. WAT is the predominant type of fat in adult humans and rodents. It not only acts as the principal storage site for lipids but also secretes adipose-derived factors (i.e. adipokines) that can affect the metabolism of other organs and tissues [10, 11]. WAT appears particularly vulnerable to suboptimal early nutrition: some of the earliest and most substantial effects of early nutritional manipulation have been observed in this tissue [12, 13]. The aims of the current study were to: (1) investigate whether changes in insulin signalling molecules in the WAT of rats exposed to a low protein diet in utero and during the suckling period are present before the development of impaired glucose tolerance; (2) determine whether these changes are cell-autonomous; and (3) further investigate the potential mechanisms involved, focusing on post-transcriptional gene regulatory mechanisms.

## Methods

### Animals

All studies were approved by the University of Cambridge Animal Welfare and Ethical Review Board, and conducted according to the Home Office Animals (Scientific Procedures, UK) Act 1986. Stock Wistar Han rats (*Rattus norvegicus*; Crl:WI[Han] strain) were purchased from Charles River (Tranent, UK) and dams were produced by in-house breeding of stock animals. Virgin female rats weighing 240–260 g were individually housed in a specific pathogen free (SPF) environment in individually ventilated cages with environmental enrichment and maintained at 22°C on a 12 h light:dark cycle. After mating, the day on which virginal plugs were expelled was taken as day 1 of gestation. Pregnant dams were given ad libitum access to control (20% protein, wt/vol.) or an isoenergetic low protein (8% protein, wt/vol.) diet [14]. At 48 h after delivery, litters were reduced at random to eight pups: four males and four females. Lactating dams were maintained on experimental diets. Twenty-one-day-old pups were weaned onto standard chow (LAD1 diet, Special Diets Services, Witham, UK), fed ad libitum and maintained in pairs in SPF cages with environmental enrichment. Two 14-month-old males from each litter were fasted overnight. The next day, their body weights were recorded, blood was taken for glucose measurement and plasma was obtained for biochemical analysis. The rats were then killed by CO_2_ asphyxiation, and individual fat depots were collected and weighed. The intra-abdominal (mesenteric) fat depot was collected from around the intestines, retroperitoneal (perirenal) fat was collected from around the kidneys and epididymal (gonadal) fat was collected from near the testes. WAT samples were snap-frozen and stored at −80°C.

### Biochemical analysis

Fasting glucose concentrations were measured in tail blood using a blood glucose analyser (AlphaTRAK, Abbott, Maidenhead, UK)). Fasting plasma insulin and leptin concentrations were determined by ELISA (ultrasensitive rat insulin EIA, Mercodia, Uppsala, Sweden; rat leptin ELISA, Crystal Chem, Downers Grove, IL, USA) and lipid content was measured using a Folch assay [15]. Lipid analysis was performed by the Core Biochemical Assay Laboratory (Cambridge, UK).

### Analysis of adipocyte cell size and number

Epididymal WAT was fixed in paraformaldehyde, processed on a 14-h cycle (VIP6, Sakula, Torrance, CA, USA), embedded in paraffin, cut into 5 μm sections, placed onto Superfrost microscope slides (VWR, Lutterworth, UK) using synthetic mountant (Thermo Scientific, Hemel Hempstead, UK) and stained with haematoxylin and eosin [16]. Images were digitally captured (at ×10 magnification) using an inverted light microscope (Olympus BX41, Olympus, Southend-on-Sea, UK). One slide was prepared for each rat and five fields of view per slide (whole cells only in field of view) were analysed and quantified using Cell^P software (Olympus, Shinjuku, Tokyo, Japan) (*n* = 6 rats per group).

### Gene expression analysis

Total RNA was extracted using Direct-zol RNA MiniPrep kits (Zymo Research, Irvine, CA, USA) with an inclusion of a DNAse digestion step. RNA purity and concentration was determined by spectrophotometric analysis (NanoDrop ND-1000, Thermo Scientific, UK), and RNA integrity was determined by agarose gel electrophoresis. cDNA was synthesised using a High-Capacity cDNA Reverse Transcription Kit (Applied Biosystems, Warrington, UK). Gene expression was assessed by quantitative (q)RT-PCR using custom designed primers (Sigma-Aldrich, Haverhill, UK and SYBR Green (Applied Biosystems; primer sequences are shown in Table 1). Analysis of melting curves confirmed the absence of primer dimers (StepOnePlus Real-Time PCR System, Applied Biosystems). Genomic rat DNA (Novagen, Madison, WI, USA) was serially diluted to generate standard curves. Several housekeeping genes were tested: *Gapdh*, *Ppia* and *Ywhaz* in epididymal fat; and *Gapdh*, *Hprt*, *Nono*, *Ppia*, *Actb*, *Ywhaz*, *Rpl* and *Gusb* in differentiated adipocytes. Expression of mRNA was normalised to *Gapdh* and *Ywhaz* expression because these genes had the least variable expression that was not affected by the maternal diet. Expression of the *Cebpa* gene (encoding CCAAT/enhancer-binding protein alpha) was determined using TaqMan gene expression assay ID Rn00560963_s1 and TaqMan reagents (Applied Biosystems).

**Table 1:** Primer sequences

Primer	Sequence (forward)	Sequence (reverse)
*Pparg*	ACACAGACAAAACATCAGTGG	ACCATGCTCTGGGTCAACAG
*Plin1*	AGGGAGGGAACCCATGGAATA	TCTTCACGCTGCAAAGCAGA
*Adipoq*	CAAGGCCGTTCTCTTCACCT	CCCCATACACTTGGAGCCAG
*Lep*	CAAAACGTGCTGCAGATAGC	CCAGCAGATGGAGGAGGTC
*Srebf1*	AAGGCCATCGACTACATCCG	TGCTTTTGTGAGCACTTCGC
*Cebpb*	TTCCTTTCCGACCTCTTCGC	CACGTAACCGTAGTCGGACG
*Cebpg*	AGTTGAGTGTGGCCTTCTCG	CGACAGCTTGCTCATTTGGG
*Insr*	TGCCCACCACCCTACTATCA	GTATAGCCAGACGGGCACTC
*Irs1*	TGGCAGTGAGGATGTGA	GGATGC TCCCCC TAGAT
*Pik3cb*	ATGGCAGACACCCTTGACAT	GGTAGCTTCCCGGGGTACTT
*Pik3r1*	AGGGGTACCAGTACAGAGCG	GTCAGGATGTCCCCCAAGTG
*Akt1*	CATGGAGTGTGTGGACAGTGA	GATGATCCATGCGGGGCTTC
*Akt2*	TGGAGCTCTGTTAGCACCGT	CAGTTCCGAGCTTGAGTGCC
*Prkcz*	CTAGCCATGGCCGGAGTG	GTCCATCTTGGGGTCGGTC
*Slc2a4*	GGCCGGGACACTATACCCTA	AGAGCCGATCTGCTGGAAAC
*Gapdh*	CAGGGCTGCCTTCTCTTGTG	GATGGTGATGGGTTTCCCGT
*Ywraz*	GGCAGAGCGATACGATGACA	AAGATGACCTACGGGCTCCT

### miRNA expression analysis

Putative miRNAs targeting the 3′ untranslated regions (UTRs) of *Irs1*, *Pik3cb* (gene encoding the catalytic subunit of PI3K [p110β]), *Pik3r1* (gene encoding the regulatory subunit of PI3K [p85α]) and *Akt1* were identified using the TargetScan [17], microRNA.org [18], miRTarBase [19] and miRanda/mirSVR [20] tools. Candidate miRNAs were ranked according to their conservation across species, the strength of the predicted interaction, whether the mRNA target site was within a proximal or distal location, and their expression in WAT. Total RNA was reverse transcribed using a TaqMan MicroRNA Reverse Transcriptase Kit (Applied Biosystems) and miRNA-specific primers (TaqMan MicroRNA Assays): miR-19a-3p, ID 000395; miR-25-3p, ID 000403; miR-30a-5p, ID 000417; miR-93-5p, ID 001090; miR-126, ID 002228; miR-128a, ID 002216; miR-130a-3p, ID 000454; miR-145, ID 002278; miR-222, ID 002276; miR-301a-3p, ID 000528; miR-320-3p, ID 002277; and miR-335-5p, ID 000546 (Applied Biosystems). qRT-PCR was performed using a TaqMan Universal PCR Master Mix No AmpErase UNG kit (Applied Biosystems). Standard curves were generated using serial dilutions of pooled sample cDNA.

### Protein analysis

Western Blotting was done as previously described [21]. Primary antibodies used were: anti-IRS-1 and anti-PI3K p85α subunit (Millipore, Lake Placid, NY, USA); anti-Akt1, anti-Akt2, anti-phospho-Akt Ser473, anti-phospho-extracellular signal-regulated kinases 1 and 2 (ERK1/2) Thr202/Tyr204, anti-phospho c-Jun N-terminal kinases 1 and 2 (JNK1/2) Thr183/Tyr185 and anti-phospho-p38 mitogen-activated protein kinase (MAPK) Thr180/Tyr182 (Cell Signaling, Danvers, MA, USA); anti-insulin receptor beta (IRβ), anti-PI3K p110β subunit, anti-protein kinase C ζ (PKCζ) and anti-suppressor of cytokine signalling 1 (SOCS1; Santa Cruz Biotechnology, Heidelberg, Germany); and anti-GLUT4 (Abcam, Cambridge, UK). Peroxidase-conjugated anti-rabbit or anti-mouse secondary antibodies were used (Jackson ImmunoResearch, Stratech, Newmarket, UK).

### Isolation and in vitro differentiation of primary pre-adipocytes

Epididymal fat was dissected and collected into Hanks Basic Salt Solution (HBSS; all chemicals from Sigma-Aldrich). For each pre-adipocyte preparation, 7 g fat was chopped, placed into 30 ml digestion solution (45 mg collagenase type II and 675 mg BSA in HBSS), shaken at 180 rev/min for 40–50 min at 37°C, strained through a 100 μm mesh (BD Falcon, Tewksbury, MA, USA) and placed on ice for 20 min. The upper layer containing mature adipocytes was then removed. The top two-thirds was mixed with growth medium (high glucose DMEM supplemented with 10% newborn calf serum, 1% penicillin-streptomycin and 200 μmol/l l-glutamine). The stromal–vascular fraction was centrifuged and re-suspended in growth medium. Following a second centrifugation, the pellets were re-suspended in 5 ml erythrocyte lysis buffer (150 mmol/l ammonium chloride, 10 mmol/l potassium bicarbonate, 0.1 mmol/l EDTA, pH 8.0) for 5 min. Cells were pelleted, re-suspended in growth medium, counted and plated at a density of 1 × 10^4^ per cm^2^ into 75 ml flasks. The culture medium was supplemented with an adipogenic cocktail (30 μmol/l insulin [Actrapid, Novo Nordisk, Gatwick, UK], 150 μmol/l sodium ascorbate) daily for the first 3 days and then every other day until harvesting on day 11.

### Statistical analysis

Data were analysed using the Student’s *t* test and corrected for multiple comparisons (Holm–Šidák method; GraphPad Prism 6, La Jolla, CA, USA). The adipocyte area is presented as median values (interquartile range [IQR]) and was analysed using the Mann–Whitney *U* test. SOCS1 data were analysed using unpaired *t-test*s with Welch’s correction. Protein content, protein phosphorylation and mRNA expression data are the percentage of control ± SEM; all other data are the means ± SEM. *n* values refer to the number of litters. A *p* value of <0.05 was considered statistically significant.

## Results

### Body weight, fat mass, fat deposition and metabolic profile

**Fig. 1:**
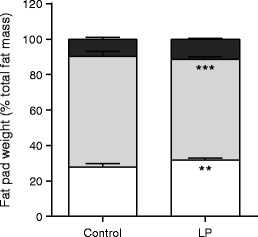
Effect of maternal low protein diet on WAT distribution in male 14-month-old offspring. The weight of epididymal (white), retroperitoneal (light grey) and intra-abdominal fat (black) in controls (*n* = 9) and LP offspring (*n* = 12) rats are presented as the percentage of total fat mass ± SEM. Data were analysed using two-way ANOVA followed by Duncan’s post hoc testing. **p < 0.01, ***p < 0.001

We confirmed previous findings of a significant lifelong reduction in body weight and length of male LP offspring compared with controls (Table 2) [22]. The retroperitoneal fat pad mass was significantly reduced in LP offspring rats in both absolute and relative terms, while no significant difference was observed in the epididymal or intra-abdominal fat pad masses (Table 2). Total fat mass (i.e. the combined weight of retroperitoneal, epididymal and intra-abdominal fat pads) was significantly reduced in LP offspring animals compared with controls in both absolute (56.5 ± 3.5 g vs 76.8 ± 5.7 g, *p* < 0.01) and relative terms (6.8% ± 0.2% vs 8.1% ± 0.4%, *p* < 0.01). To investigate WAT distribution, we calculated the weight of individual fat pads as the percentage of total fat mass (Fig. 1). Two-way ANOVA revealed an interaction between maternal diet and fat distribution (*p* = 0.00001), with the LP offspring group showing a relative increase in epididymal (*p* < 0.01) and reduction in retroperitoneal (*p* < 0.001) fat pad masses compared with controls (Fig. 1).

**Table 2:** Body weight and body length, absolute and relative fat pad weights

Characteristic	Control offspring	LP offspring
Body weight (g)	946 ± 47	813 ± 30*
Body length (cm)	291 ± 2	281 ± 2**
Epididymal fat (g)	19.8 ± 0.9	17.5 ± 0.9
Epididymal fat (% BW)	2.1 ± 0.1	2.2 ± 0.0
Retroperitoneal fat (g)	50.4 ± 4.9	32.7 ± 2.6**
Retroperitoneal fat (% BW)	5.3 ± 0.4	3.9 ± 0.2**
Intra-abdominal fat (g)	6.6 ± 0.4	6.2 ± 0.4
Intra-abdominal fat (% BW)	0.7 ± 0.0	0.8 ± 0.0

Data are means ± SEM

*p < 0.05, **p < 0.01

BW, body weight

Fasting glucose, insulin, leptin, cholesterol, triacylglycerol, HDL and NEFA levels were not different between the two groups (Table 3). This supports our strategy of investigating molecular changes that precede any differences in metabolic status.

**Table 3:** Metabolic profile of 14-month-old control and LP offspring

Metabolic factor	Control offspring	LP offspring
Glucose (mmol/l)	5.5 ± 0.2	5.5 ± 0.2
Insulin (pmol)	270 ± 28	248 ± 31
Leptin (ng/μl)	39 ± 7	38 ± 7
Cholesterol (mmol/l)	6.23 ± 1.24	5.43 ± 0.55
Triacylglycerols (mmol/l)	0.7 ± 0.2	0.5 ± 0.1
HDL (mmol/l)	1.70 ± 0.44	1.37 ± 0.16
NEFA (μmol/l)	575 ± 62	618 ± 46

Data are the means ± SEM. Due to the unequal variances, data for triacylglycerols was log_10_-transformed prior to the analysis

### Morphology of epididymal WAT

**Fig. 2:**
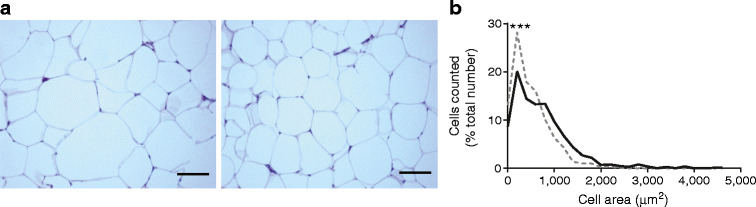
Effect of maternal low protein diet on the epididymal adipose tissue morphology of 14-month-old offspring rats. (**a**) Representative histological images from controls (left) and LP offspring (right). Scale bar represents 100 μm. (**b**) Adipocyte area frequency distribution of control (black line) and LP offspring (dashed line) rats. Median adipocyte area within epididymal adipose tissue differed between LP offspring and controls (*p* < 0.001, Mann–Whitney *U* test; *n* = 6 rats per group). One slide was prepared from each rat and five fields of view were analysed per slide. ***p < 0.001

The median adipocyte area was reduced in epididymal WAT from LP offspring compared with controls (387.2 μm^2^ [IQR 176.6–696.5 μm^2^] vs 600.7 μm^2^ [IQR 260.2–1,004.0 μm^2^], *p* < 0.001; Mann–Whitney *U* test). Epididymal WAT from LP offspring contained a higher percentage of small adipocytes compared with controls (*p* < 0.001; Fig. 2). However, lipid content was comparable between the two groups (controls: 70.1% ± 2.0% vs LP offspring: 72.3% ± 2.1% of total weight of a fat pad).

### Insulin signalling proteins in epididymal fat

**Fig. 3:**
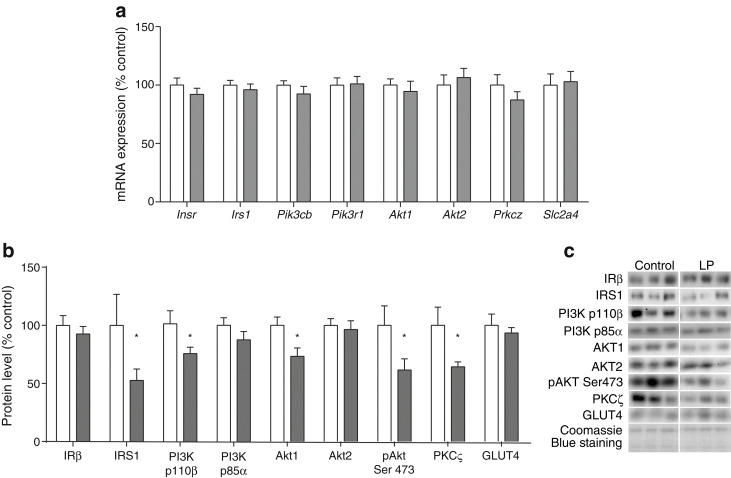
Effect of maternal low protein diet on the expression of insulin signalling molecules in epididymal WAT of 14-month-old male offspring rats. (**a**) mRNA levels, (**b**) protein content and phosphorylation and (**c**) representative protein blots. (**a**, **b**) Data are the percentage of control values ± SEM; *n* = 8 for controls, *n* = 10 for LP offspring; *p < 0.05. White bars, control; grey bars, LP. *Slc2a4* encodes GLUT4

No differences in mRNA expression were observed for key insulin signalling molecules in epididymal WAT (Fig. 3a). IRβ protein levels were not different between the two groups (Fig. 3b). However, the protein content of downstream signalling molecules including IRS1 and the PI3K catalytic subunit p110β were significantly reduced in epididymal WAT from LP offspring (*p* < 0.05 for both). Levels of PI3K regulatory subunit p85α were comparable to those of controls (Fig. 3b). Akt1 protein was significantly reduced in LP offspring (*p* < 0.05), while Akt2 remained unchanged. Phosphorylated Akt (Ser473) and PKCζ protein levels were also decreased in LP offspring (both *p* < 0.05), while levels of GLUT4 were comparable to those of controls (Fig. 3a). These findings are consistent with WAT insulin resistance in the LP offspring.

### Levels of key insulin signalling proteins are reduced in in vitro differentiated pre-adipocytes from LP offspring

**Fig. 4:**
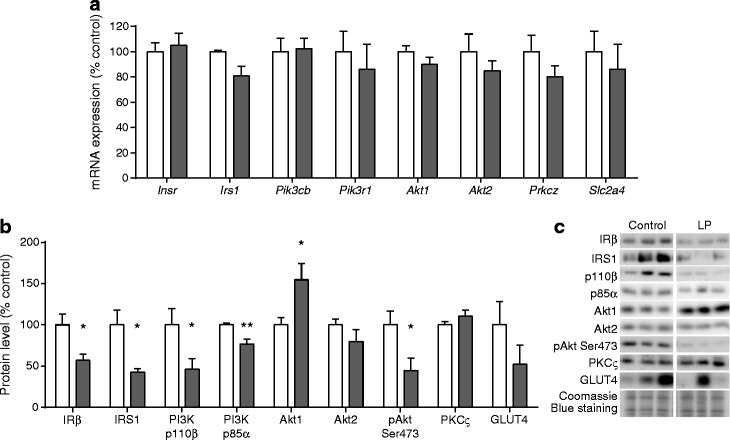
Effect of maternal low protein diet on the levels of key insulin signalling molecules in differentiated adipocytes from offspring rats. (**a**) mRNA levels in controls (*n* = 8) and LP offspring (*n* = 10). (**b**) Protein content and phosphorylation (*n* = 6 per group). (**c**) Representative protein blots. (**a**, **b**) Data are the percentage of control values ± SEM; *p < 0.05, **p < 0.01. White bars, control; grey bars, LP. *Slc2a4* encodes GLUT4

There was no difference in the mRNA level of any of the key insulin signalling molecules studied in adipocytes derived from LP offspring and control pre-adipocytes after in vitro differentiation (Fig. 4a). However, levels of IRβ (*p* < 0.05), IRS1 (*p* < 0.05), PI3K p110β (*p* < 0.05) and PI3K p85α (*p* < 0.01) proteins were significantly reduced in differentiated adipocytes derived from LP offspring compared with controls (Fig. 4b). Despite the increase in Akt1 protein level (*p* < 0.05) and no change in Akt2 protein level, Akt Ser473 phosphorylation was also reduced (*p* < 0.05) in in vitro differentiated adipocytes from LP offspring (Fig. 4b). This suggests reduced insulin action in programmed adipocytes after in vitro differentiation.

### Effect of maternal protein restriction on pre-adipocyte differentiation

We next investigated whether the reduced protein content and phosphorylation of key insulin signalling proteins in differentiated adipocytes from LP offspring were related to differences in the levels of differentiation markers. As differentiation is mainly driven by transcriptional mechanisms, we measured the mRNA expression of key differentiation marker genes: *Pparg*, *Plin1*, *Adipoq*, *Lep*, *Srebf1* (gene encoding sterol regulatory element-binding protein 1), *Cebpa*, *Cebpb* and *Cebpg*. We did not observe differences in the mRNA levels of any of these markers (Table 4), suggesting that impaired insulin signalling and action in LP offspring adipocytes is not related to differences in differentiation.

**Table 4:** Levels of differentiation marker mRNAs in in vitro differentiated adipocytes

mRNA	Control offspring	LP offspring	*p* value
*Pparg*	0.014 ± 0.001	0.012 ± 0.001	0.22
*Plin1*	0.181 ± 0.033	0.169 ± 0.046	0.81
*Adipoq*	1.550 ± 0.300	1.753 ± 0.416	0.72
*Lep*	0.140 ± 0.027	0.109 ± 0.016	0.30
*Srebf1*	0.670 ± 0.138	0.497 ± 0.101	0.31
*Cebpa*	0.084 ± 0.014	0.060 ± 0.012	0.12
*Cebpb*	1.389 ± 0.096	1.176 ± 0.095	0.15
*Cebpg*	0.031 ± 0.003	0.026 ± 0.002	0.19

Data are the means ± SEM

### MiRNA expression in primary pre-adipocytes after in vitro differentiation

**Fig. 5:**
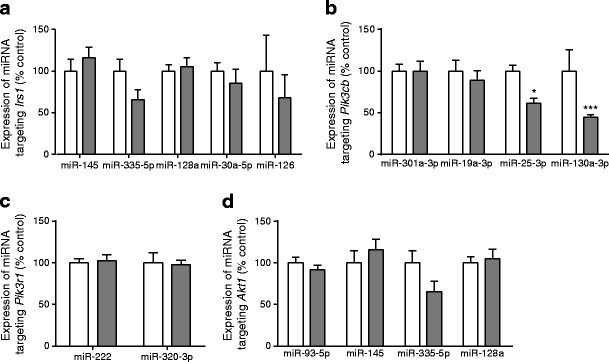
The effect of maternal low protein diet on expression of miRNAs targeting the mRNA 3′UTRs of key insulin signalling genes in differentiated adipocytes from offspring rats. (**a**) *Irs1*, (**b**) *Pik3cb*, (**c**) *Pik3r1*, (**d**) *Akt1*. Data are the percentage of control values ± SEM; *n* = 8 controls, *n* = 10 LP offspring; *p < 0.05, ***p < 0.001. White bars, control; grey bars, LP

As a maternal low protein diet had profound effects on the levels of IRS1, PI3K p85α and p110β subunits, and Akt1 without changing the corresponding mRNA levels, we investigated the mechanisms underlying these effects with a particular focus on the potential role of miRNAs. We used a candidate approach to examine the expression of miRNAs with complementary binding seed sequences in the 3′UTR of four genes: *Irs1*, *Pik3cb*, *Pik3r1* and *Akt1*. For each gene, a panel of miRNAs was selected following bioinformatic assessment. None of the miRNAs selected for potentially regulating *Irs1*, *Pik3r1* or *Akt1* were upregulated in LP offspring (Fig. 5). Of the four miRNAs selected for *Pik3cb*, expression of two, miR-25-3p and miR-130a-3p, was reduced (*p* < 0.05 and *p* < 0.001, respectively) in in vitro differentiated adipocytes from LP offspring (Fig. 5).

### Markers of protein degradation in in vitro differentiated pre-adipocytes from LP offspring

**Fig. 6:**
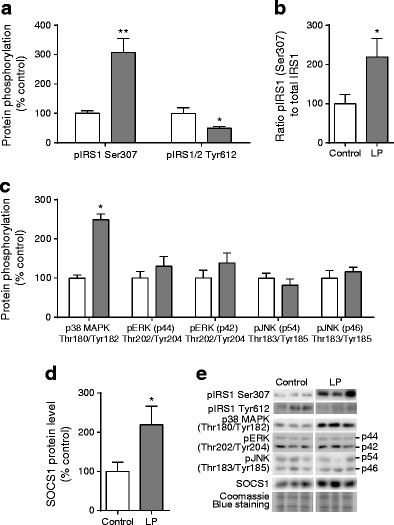
The effects of maternal low protein diet on levels of markers of protein degradation in differentiated adipocytes from offspring rats. (**a**) IRS1 Ser307 and Tyr612 phosphorylation (pIRS1, phospho-IRS1), (**b**) ratio of Ser307 pIRS1 to total IRS1, (**c**) MAP kinase phosphorylation and (**d**) SOCS1 protein levels. (**e**) Representative protein blots. Data are the percentage of control values ± SEM; *n* = 6 per group. White bars, control; grey bars, LP. pERK, phospho-ERK; pJNK, phospho-JNK

Maternal protein restriction led to a significant increase in IRS1 Ser307 phosphorylation in differentiated adipocytes from LP offspring (*p* < 0.01), while IRS1 Tyr612 phosphorylation was reduced (*p* < 0.05; Fig. 6a). The ratio of Ser307 phosphorylated IRS1 to total IRS1 was increased in differentiated adipocytes from LP offspring compared with controls (*p* < 0.001; Fig. 6b). There was no difference in ERK1/2 Thr202/Tyr204 phosphorylation or JNK1/2 Thr183/Tyr185 phosphorylation between the groups; however, p38 MAPK Thr180/Tyr182 phosphorylation was increased in differentiated adipocytes from LP offspring animals (*p* < 0.05; Fig. 6c). SOCS1 protein content was also increased in differentiated adipocytes from LP offspring compared with controls (*p* < 0.05; Fig. 6d).

## Discussion

The present study investigated the mechanisms involved in mediating the effects of suboptimal early nutrition on the development of peripheral insulin resistance. Significant changes in body fat distribution, adipocyte size and the levels and phosphorylation status of key insulin signalling proteins were observed in WAT from adult male rats exposed to maternal protein restriction during gestation and lactation. Moreover, the molecular phenotype of impaired insulin signalling was retained in isolated pre-adipocytes from LP offspring after in vitro differentiation. Importantly, as LP offspring were normoglycaemic and normoinsulinaemic at the time of study, these changes are not a consequence of metabolic dysfunction but are likely to contribute to the later development of whole body insulin resistance and type 2 diabetes.

We observed that maternal protein restriction led to a reduction in total visceral WAT (intra-abdominal, retroperitoneal and epididymal fat depots combined) and altered the distribution of fat within the body in the offspring. In LP offspring, relative fat deposition within the epididymal depot was increased, while deposition into the retroperitoneal depot was reduced. Changes in body fat distribution have been observed in young, lean normoglycaemic LBW men, and may contribute to the development of impaired glucose tolerance and type 2 diabetes in developmentally programmed individuals [23]. Altered fat deposition may lead to alterations in sympathetic drive and rates of lipolysis because these vary between different fat depots [10]. Increased rates of lipolysis have been found in adipocytes isolated from 3-month-old LP offspring rats and LBW men [24–26]. However, the full impact of altered fat distribution in LP offspring on metabolic phenotype remains to be determined.

We have previously shown that 3-month-old LP offspring have reduced adipocyte size. This was associated with increased miR-483 expression in both rodents and humans and led to a reduction in the expandability of adipose tissue [7]. Here, we showed that reduced adipocyte size is retained in 14-month-old LP offspring rats and that a higher percentage of small adipocytes can be found in epididymal WAT of these animals compared with controls. An increased proportion of small adipocytes and a corresponding deficit in large adipocytes have been associated with insulin resistance [27].

Impairments in insulin signalling including decreased IRS1 protein content, reduced Akt Ser473 phosphorylation, impaired insulin-stimulated PI3K activity and impaired glucose transport have been observed in adipocytes from individuals with a predisposition toward type 2 diabetes (i.e. with at least two first-degree relatives diagnosed with diabetes) [28]. A similar pattern of abnormalities can be detected in adipocytes from diabetic patients and LBW young men [5]. Here, we show reduced levels of IRS1, the p110β catalytic subunit of PI3K, Akt1 and PKCζ, and decreased Akt Ser473 phosphorylation in the epididymal fat of LP offspring. Importantly, we demonstrate that the reduction in key insulin signalling proteins (IRβ, IRS1, PI3K p85α and p110β subunits and Akt1) and decreased Akt Ser473 phosphorylation is retained in isolated primary pre-adipocytes from LP offspring after in vitro differentiation. This strongly suggests that cell-autonomous mechanism(s) retained throughout the life-course underlie the programmed phenotype. These are maintained following multiple rounds of cell division and differentiation in a controlled environment. As individuals with heritable predisposition to diabetes, LBW humans, LP offspring rats and mice exposed to maternal obesity during gestation and lactation had similar impairments in insulin signalling proteins in WAT (with IRS1 downregulation in mice also shown to be regulated in a cell-autonomous manner), common pathways may be initiated in response to a range of suboptimal early conditions that may drive the development of peripheral insulin resistance [2, 5, 8, 28].

Although until recently research in the programming field has been primarily directed at understanding mechanisms mediating changes in gene expression at a transcriptional level, changes in the levels of multiple proteins are seen without alterations in the corresponding mRNA transcript levels [7, 8, 12]. Our finding in LP offspring of impaired insulin signalling protein levels in both WAT and adipocytes in vitro differentiated without changes in the corresponding mRNA levels implies that the mechanisms underlying these programmed changes occur at the post-transcriptional level, and involve changes in protein synthesis and/or degradation.

To investigate the cell-autonomous mechanisms that may mediate the effects of maternal nutrition on susceptibility to developing peripheral insulin resistance in WAT in the offspring, we studied miRNAs implicated in regulating the synthesis of programmed insulin signalling proteins. MiRNAs repress mRNA translation by binding to specific sequences within the 3′UTRs of target mRNAs. Although numerous miRNAs have been implicated in the pathogenesis of insulin resistance and type 2 diabetes [29], information on which miRNAs may be involved in developmental programming in WAT is scarce. Recently, a programmed increase in miR-126, was shown to repress translation of its *Irs1* target in WAT from mice exposed to maternal overnutrition in early life [8]. Here, we used a candidate approach to identify miRNAs that could potentially influence the translation of *Irs1*, *Pik3cb*, *Pik3r1* and *Akt1* mRNAs. However, none of the candidate miRNAs (including miR-126) were upregulated in adipocytes that were differentiated in utero in response to maternal low protein diet. Therefore, none of the changes in the levels of insulin signalling molecules could be explained by upregulation of these miRNAs. This implies that: (1) altered levels of these proteins are not mediated by the candidate miRNAs studied; (2) other unidentified miRNAs play a role; or (3) other post-transcriptional mechanisms are involved.

In the current study, we observed increased serine phosphorylation and decreased tyrosine phosphorylation in IRS1 in in vitro differentiated adipocytes from LP offspring. Although tyrosine phosphorylation is required to generate the insulin signal, serine phosphorylation occurs before, during and after insulin stimulation [30]. Increased IRS1 phosphorylation at serine 307 (rodents)/312 (humans) has been reported in insulin resistant states [9, 31] and nutritionally programmed animals [21]. One mechanism through which IRS1 can become phosphorylated at Ser307 involves activation of JNK1/2, a key component of the stress and inflammatory response pathway [32]. However, we observed no differences in JNK1/2 Thr183/Tyr185 phosphorylation in differentiated adipocytes from LP offspring and controls, suggesting that other cell-autonomous mechanism(s) must be involved in upregulating IRS1 Ser307 phosphorylation. Prolonged IRS1 serine/threonine phosphorylation following chronic insulin stimulation leads to its targeted degradation by the proteasome [33, 34]. Increased IRS1 ubiquitination and its subsequent degradation have been reported in a mouse model of type 2 diabetes (TALLYHO/Jng mice) and were associated with an increase in SOCS1 levels [9]. SOCS proteins are important mediators of IRS1 degradation and target IRS1 via interaction with the SOCS box motif [35]. Increased levels of SOCS1 in differentiated adipocytes from LP offspring animals suggests that lower IRS1 protein levels in these animals may be at least partly caused by SOCS1-mediated degradation of IRS1.

We investigated whether the other two MAPKs could play a role in the cell-autonomous programming of insulin signalling proteins. There was no difference in ERK Thr202/Tyr204 phosphorylation in differentiated adipocytes from LP offspring and controls. However, p38 MAPK Thr180/Tyr182 phosphorylation was significantly upregulated in differentiated adipocytes from LP offspring animals. Enhanced basal p38 MAPK activation has also been reported in adipocytes from type 2 diabetic patients [36]. An in vitro study of isolated skeletal muscle strips showed that low levels of oxidative stress lead to increased p38 MAPK Thr180/Tyr182 phosphorylation which was associated with decreased IRS1 protein levels and inhibition of insulin-stimulated Akt Ser473 phosphorylation [37].

In conclusion, we have demonstrated that a maternal low protein diet during gestation and lactation leads to altered adipose tissues distribution and insulin resistance in the WAT of male offspring. This is associated with reductions in protein levels and phosphorylation of key insulin signalling molecules in WAT. This phenotype was retained in primary in vitro differentiated adipocytes, suggesting that cell-autonomous mechanism(s) are responsible. These findings bring us a step closer to understanding the mechanisms that mediate the effects of suboptimal early nutrition on the risk of developing type 2 diabetes.

## Abbreviations

ERK: Extracellular signal-regulated kinase
GLUT4: Glucose transporter type 4
IRβ: Insulin receptor beta
JNK: c-Jun N-terminal kinase
LBW: Low birthweight
LP offspring: Offspring of low protein fed dams
MAPK: Mitogen-activated protein kinase
MiRNA: microRNA
PI3K: Phosphatidylinositol 3-kinase
PKCζ: Protein kinase Cζ
qRT-PCR: Quantitative RT-PCR
SOCS1: Suppressor of cytokine signalling 1
UTR: Untranslated region
WAT: White adipose tissue
