# Correction to: Cell-autonomous programming of rat adipose tissue insulin signalling proteins by maternal nutrition

**DOI:** 10.1007/s00125-023-06036-w

**Published:** 2023-11-06

**Authors:** Malgorzata S. Martin-Gronert, Denise S. Fernandez-Twinn, Martin Bushell, Kenneth Siddle, Susan E. Ozanne

**Affiliations:** 1 University of Cambridge Metabolic Research Laboratories and MRC Metabolic Diseases Unit, Wellcome Trust—MRC Institute of Metabolic Science, Addenbrooke’s Hospital, Box 289, Cambridge, CB2 OQQ UK; 2 MRC Toxicology Unit, University of Leicester, Hodgkin Building, Leicester, UK

**Correction to: Diabetologia**

**https://doi.org/10.1007/s00125-016-3905-8**

In the western blot shown in Fig. 3c, the representative image used in the IRβ panel for the LP group was a duplication of the image used for the control panel. The authors assert that this mistake had no impact on the data analysis, interpretation or conclusions drawn, including the band densitometry shown in Fig. 3b. Figure 3c in the original article has been corrected.

**Fig. 3:**
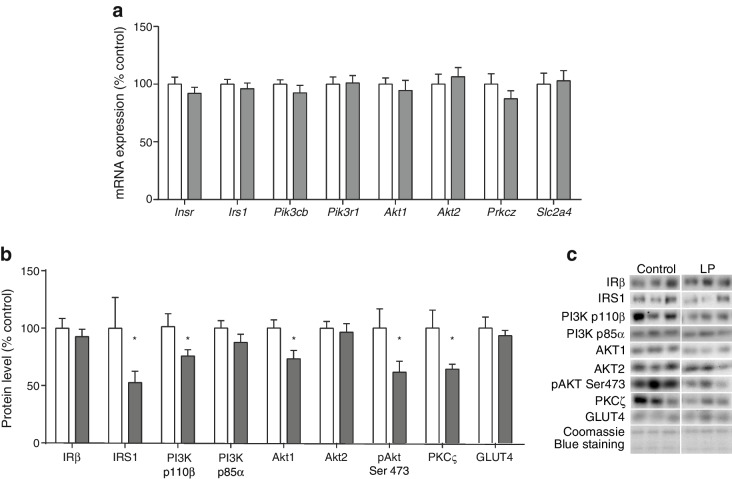
Effect of maternal low protein diet on the expression of insulin signalling molecules in epididymal WAT of 14-month-old male offspring rats. (**a**) mRNA levels, (**b**) protein content and phosphorylation and (**c**) representative protein blots. (**a**, **b**) Data are the percentage of control values ± SEM; *n* = 8 for controls, *n* = 10 for LP offspring; *p < 0.05. White bars, control; grey bars, LP. *Slc2a4* encodes GLUT4

